# Characterization of the reproductive tract bacterial microbiota of virgin, mated, and blood-fed *Aedes aegypti* and *Aedes albopictus* females

**DOI:** 10.1186/s13071-021-05093-7

**Published:** 2021-12-01

**Authors:** Sebastián Díaz, Carolina Camargo, Frank W. Avila

**Affiliations:** Max Planck Tandem Group in Mosquito Reproductive Biology, Universidad de Antioquia, Medellín, 050010 Antioquia Colombia

**Keywords:** *Aedes aegypti*, *Aedes albopictus*, Reproductive tract, Microbiota, Mating, Blood-feeding

## Abstract

**Background:**

*Aedes aegypti* and *Ae. albopictus* are vectors of numerous arboviruses that adversely affect human health. In mosquito vectors of disease, the bacterial microbiota influence several physiological processes, including fertility and vector competence, making manipulation of the bacterial community a promising method to control mosquito vectors. In this study, we describe the reproductive tract tissue microbiota of lab-reared virgin *Ae. aegypti* and *Ae. albopictus* males, and virgin, mated, and mated + blood-fed females of each species, comparing the bacterial composition found there to the well-described gut microbiota.

**Methods:**

We performed metabarcoding of the 16S rRNA isolated from the gut, upper reproductive tract (URT; testes or ovaries), and lower reproductive tract (LRT; males: seminal vesicles and accessory glands; females: oviduct, spermathecae, and bursa) for each species, and evaluated the influence of host species, tissue, nutritional status, and reproductive status on microbiota composition. Finally, based on the identified taxonomic profiles of the tissues assessed, bacterial metabolic pathway abundance was predicted.

**Results:**

The community structure of the reproductive tract is unique compared to the gut. *Asaia* is the most prevalent OTU in the LRTs of both *Ae. aegypti* and *Ae. albopictus*. In the URT, we observed differences between species, with *Wolbachia* OTUs being dominant in the *Ae. albopictus* URT, while *Enterobacter* and *Serratia* were dominant in *Ae. aegypti* URT. Host species and tissue were the best predictors of the community composition compared to reproductive status (i.e., virgin or mated) and nutritional status (i.e., sugar or blood-fed). The predicted functional profile shows changes in the abundance of specific microbial pathways that are associated with mating and blood-feeding, like energy production in mated tissues and siderophore synthesis in blood-fed female tissues.

**Conclusions:**

*Aedes aegypti* and *Ae. albopictus* have distinct differences in the composition of microbiota found in the reproductive tract. The distribution of the bacterial taxonomic groups indicates that some bacteria have tissue-specific tropism for reproductive tract tissue, such as *Asaia* and *Wolbachia*. No significant differences in the taxonomic composition were observed in the reproductive tract between virgin, mated, and mated + blood-fed females, but changes in the abundance of specific metabolic pathways were found in the predicted microbial functional profiles in mated and blood-fed females.

**Graphical Abstract:**

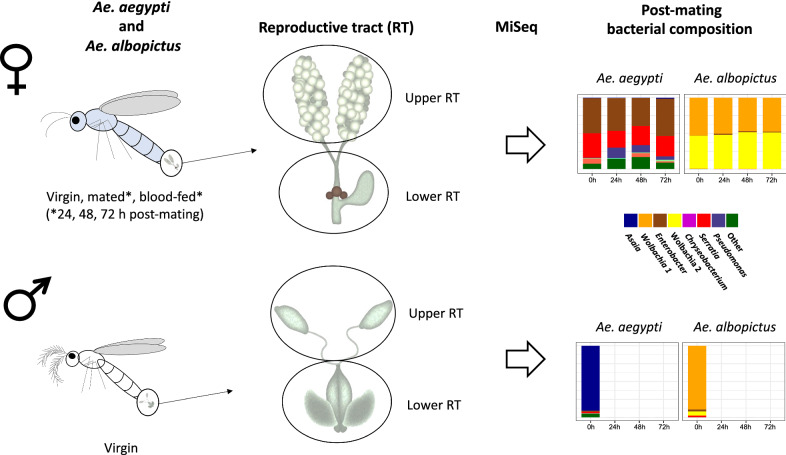

**Supplementary Information:**

The online version contains supplementary material available at 10.1186/s13071-021-05093-7.

## Background

*Aedes aegypti* and *Ae. albopictus* are invasive mosquito species that are responsible for the dissemination of numerous viruses that adversely affect human health, including dengue [1], Zika [2], and chikungunya [3] viruses. These species are found throughout the tropics and subtropics, and their populations often overlap [4], with *Ae. albopictus* further distributed in temperate regions, as this species can tolerate colder temperatures and diapause during winter months [5–7]. The predicted territories of both *Ae. aegypti* and *Ae. albopictus* are expected to expand with increasing global temperatures [8], making the development of novel control strategies essential to minimize the impact of the diseases transmitted by these vectors.

The expansion of territory habitable by *Aedes* vectors requires their successful reproduction. After mating, *Aedes* females undergo numerous physiological and behavioral changes that primarily act to facilitate the production of progeny. Male-derived seminal fluid proteins, transferred to females along with sperm during mating, induce several of the observed post-mating changes in *Aedes* mosquitoes [9, 10], although female contributions are also likely to be involved as in other insects [11]. While male-derived gene products are essential for fertility, other factors can alter the fertility of female insects, such as nutritional status [12, 13], immune function [14], male age [15], body size [16, 17], and temperature [18]. Another factor that can alter fertility is the composition of bacterial microbiota [19–21].

Most of the mosquito bacterial microbiota is acquired from the aquatic larvae habitat [22, 23] or from nectar-feeding by adult mosquitos [24], although some bacteria can be transmitted vertically, such as *Wolbachia* and *Asaia* [25]. Bacterial microbiota influence several physiological processes including larval growth, blood-digestion rates, and immune function [26–28]. To date, most studies that have examined the bacterial composition of adult tissues have focused on the gut. However, mosquito bacterial microbiota is not exclusive to the gut, having been described in other tissues such as the salivary glands [28]. Studies that have examined the composition of reproductive tract (RT) microbiota are limited [29–31], with one reporting microbiota from *Ae. aegypti* and *Ae. albopictus* RT tissues [31], and another sampling the cultivable bacteria of *Ae. aegypti* ovaries [32]. The identification of the microbiota composition of male and female *Aedes* RT tissues is a first step in understanding how these communities interact with host fertility.

In anautogenous mosquitoes, reproduction requires a successful copulation and ingestion of a blood meal to acquire nutrients for egg development. The seminal fluid of both *Ae. aegypti* and *Ae. albopictus* contain proteins with immunity-related functions [33–35], and mating increases the expression of immunity-related genes in the *Ae. aegypti* female RT, including genes that code for defensins, transferrin, cecropins, lectins, and a Kazal-type serine protease inhibitor [36–38]. Although blood consumption by female mosquitoes induces oxidative stress due to the release of iron during hemoglobin digestion [39], blood ingestion alters the microbiota structure of the gut, reducing diversity but favoring proliferation of enteric bacteria [40]. In *Ae. aegypti* females, the proliferation of gut microbiota is observed after a blood meal [41]. How mating and/or blood-feeding may influence the RT tissue microbiota community has not been examined.

Microbiota can also influence physiological processes and behaviors in adult insects important in reproduction, suggesting that specific bacteria may influence host fertility. For example, microbial symbionts can alter host pheromone profiles [42], potentially influencing mate localization and mate choice. The post-mating transcriptome of *Drosophila melanogaster* females is altered by the microbiome [21], suggesting that bacteria may affect downstream processes elicited by seminal protein receipt. In adult mosquitoes, microbiota composition influences mating preferences [29] and fertility [43, 44]. Recently, *Serratia* infection of female *Ae. aegypti* was shown to alter their blood-feeding propensity [45]. Further, microbiota prevalence is also influenced by microbial interactions [45–47]. The identification of *Ae. aegypti* and *Ae. albopictus* RT tissue-associated microbiota may highlight microbiota that influence reproductive parameters in these vector species.

To gain insight into the bacterial diversity and their potential roles in RT tissues, we described the bacterial community of males and females from laboratory populations of *Ae. aegypti* and *Ae. albopictus* reared under identical conditions. Our goal was to identify the microbiota composition of the upper and lower reproductive tracts (URT and LRT, respectively) in virgin males and females, and in mated and blood-fed females of each species (URT: testes or ovaries; LRT males: seminal vesicles and accessory glands; females: oviduct, spermathecae, and bursa). Additionally, we assessed how the composition of RT tissues compared to the gut microbiota. Finally, we used the predicted metabolic abundance profile to evaluate the interaction of the microbiota physiology with the biological changes of the host.

## Methods

### Mosquitoes

Thai strain *Ae. aegypti* was collected in Bangkok, Thailand, and has been maintained in colony since 2009. The *Ae. albopictus* strain was established in 2018 from eggs oviposited in ovitraps in Medellín, Colombia. Eggs were hatched under vacuum pressure (−50 kPa) for 30 min in 250 ml double-distilled water (ddH_2_O) supplemented with a pinch of active yeast. The resulting larvae were allocated into rearing trays 24 h later at a density of 200 per tray in 1 L ddH2O and fed four Hikari Gold Cichlid food pellets (Hikari, Himeji, Japan). Pupae were separated into 5 ml vials to ensure virginity. Adults were transferred to single-sex 8 L cages upon eclosion. Larvae, pupae, and adults were maintained in an environmental chamber at 27 °C, 80% relative humidity, and a 12/12 light/dark cycle, and had constant access to 10% sucrose.

### Mating and sample preparation

Four- to 6-day-old adults were used in our experiments. We observed all matings by placing one female and three males into an 8 L cage until copulation occurred, defined as engagement of male–female genitalia for ≥ 10 s for *Ae. aegypti* and ≥ 30 s for *Ae. albopictus*; these parameters result in successful insemination in 99% of *Ae. aegypti* and 91% of *Ae. albopictus* [48, 49]. Males were subsequently discarded. Females were grouped into 20 min mating intervals. A subset of females were blood-fed on the arm of a volunteer following a mating interval; only engorged females were considered blood-fed. Blood-feeding was approved by the Human Research Bioethics Committee (Universidad de Antioquia), and all volunteers signed a consent form. Females were housed in 500 ml cups and maintained in an environmental chamber until they were flash-frozen at the appropriate time point. Specimens were stored at −80 °C until tissue dissection.

We used the following treatments: virgin males and females frozen on the same day as the matings, mated females, non-blood-fed females at 0 h (frozen immediately after the 20 min mating interval), 24, 48, and 72 h post-mating, and mated + blood-fed females at 24, 48, and 72 h post-mating. Virgin females from the same cohort were also collected at each time point (24, 48, and 72 h) as an age control. For tissue samples, the midgut, URT (female: ovaries; male: testes), and LRT (female: oviduct, spermathecae, spermathecal vestibule, and bursa; male: vas deferens, accessory glands, and seminal vesicle) were dissected in 1× phosphate-buffered saline (PBS) under sterile conditions. Pools of 20 tissues per treatment were stored in STE buffer (100 mM NaCl, 10 mM Tris–Cl, pH 8.0, and 1 mM EDTA) at −80 °C until DNA extraction.

For both species, samples were also extracted from (1) whole-body male and female adults (a pool of three virgin individuals), (2) larvae rearing water from the final pupating day (merging aliquots from all rearing containers to a final volume of 1.5 ml), (3) freshly prepared 10% sucrose solution fed to adults, (4) 10% sucrose solution sampled again on the day before mating (merging aliquots from all the cages to a final volume of 1.5 ml), (5) a larval food pellet (200 mg), (6) a 1× PBS blank control (1.5 ml), and (7) DNA extraction reagents controls, one for each extraction round for a total of five samples.

Samples were randomly seeded into five different extraction days. Liquid samples (i.e., larvae water, sucrose solution, and 1 × PBS) were centrifuged at 13,000 rpm for 1 min and the supernatant discarded. For each sample, lysis was started by adding 6 µl of lysozyme (20 µg/µl) for 2 h at 37 °C, followed by an overnight incubation adding 24 µl of proteinase K (20 µg/µl) at 56 °C. DNA was extracted using a phenol–chloroform protocol and eluted in 50 µl of Buffer AE (Qiagen, Valencia, CA, USA). Samples were quantified using the Qubit dsDNA BR Assay Kit (Life Technologies, Carlsbad, CA, USA). A diagnostic polymerase chain reaction (PCR) was performed to evaluate the quality of bacterial DNA using the P338F (5′-ACT CCT ACG GGA GGC AGC AG-3′) and 1492R (5′-NTA CCT TGT TAC GAC T-3′) primers that targeted the 16S rRNA gene. Positive tissue samples and controls were sent to Macrogen (Seoul, Korea) for sequencing on the Illumina platform using the primers Bakt_341F (5′-CCT ACG GGN GGC WGC AG-3′) and Bakt_805R (5′-GAC TAC HVG GGT ATC TAA TCC-3′) [50], which correspond to the V3-V4 hypervariable regions of the 16S rRNA gene with a sequencing depth of 100,000 reads per sample.

### Metabarcoding analysis

Raw reads were processed with Mothur v. 1.43.0 [51]. Low-quality sequences with (1) the presence of ambiguous nucleotides, (2) more than eight homopolymers, (c) sequence length lower than the 2.5th percentile, and (4) sequence length higher than the 97.5th percentile, were filtered out. The remaining sequences were pre-clustered to reduce sequencing error (allowing one difference for every 100 base pairs [bp] of sequence). Chimeras were removed with VSEARCH [52]. Non-bacterial sequences were removed based on a preliminary classification using the SILVA v132 database [53]. Singletons were removed to avoid operational taxonomic unit (OTU) overestimation and because of the high sequencing depth of the sampling. Samples were normalized to 25,000 sequences.

To decrease the effect of contamination, we initially used a clustering-free approach to define our OTUs [54]. After cleaning the dataset, each unique sequence (i.e., 100% nucleotide identity) was defined as an amplicon sequence variant (ASV). We looked for ASVs shared between the negative controls (1× PBS and DNA extraction reagents samples) and tissue samples. To avoid removing false-negative sequences due to cross-contamination between samples and negative controls [54], we only removed ASVs with an abundance of ≥ 1% in the negative control samples—four in total (two *Enterobacter*, one *Serratia,* and one *Cutibacterium*). Using the remaining sequences, we clustered OTUs at a 97% identity level using the OptiClust algorithm implemented in Mothur [54]. We calculated the sampling effort with rarefaction curves and sample coverage index (Good's coverage), and alpha diversity metrics for richness (number of OTUs), diversity (Shannon index), and evenness (Pielou’s index).

An indicator species analysis, to evaluate OTUs that were strongly associated with a specific experimental variable, was performed [55] using the labdsv package [56] implemented in R (http://www.r-project.org/); indicator values range from 0 to 1.0, with higher values indicating OTU specificity for treatment. We evaluated (1) host species, (2) sampled tissues, (3) mating status (virgin or mated), and (4) nutritional status (non-blood-fed or blood-fed). To quantify the overall influence of the experimental variables (the same as the indicator species analysis plus time points) in the bacterial community composition, we performed a permutational analysis of variance (PERMANOVA) [57] in the vegan package [58] also implemented in R. To complement this analysis, we performed an ordination analysis. First, we evaluated a linearity assumption using a detrended canonical correspondence analysis (DCA). As our samples did not meet this criterion, a canonical correspondence analysis (CCA) was implemented as an ordination method.

We used PICRUSt 2.0 [59] to analyze the predicted bacterial functional profile. Based on the OTU taxonomy and relative abundance for each sample, the biological pathways used for the bacterial community were predicted and classified according to the MetaCyc database [60]. Metabolic pathways with low abundance values were filtered from the matrix (counts per million [CPM] < 1) [61] to identify sources of erroneous variation, which were included in a design matrix that was entered into edgeR [62]. We used a range of biological coefficient of variation (BCV) estimates to extract significant different metabolic pathways from this data, and ultimately found that a value of 0.1 gives a conservative value to develop a differential testing. To perform metabolic pathway-wise differential testing, we used a fit generalized linear model and ultimately performed an *F*-test to identify differentially abundant metabolic pathways compared to a baseline sample. For both species, we evaluated which metabolic pathways were significantly different after (1) mating, using virgin females as the baseline samples compared to mated, non-blood-fed females; and (2) blood-feeding, using mated non-blood-fed females as the baseline samples compared to the blood-fed at the same post-mating time point. Metabolic predictions were performed in the samples of two time points of special interest in the host physiology: 24 h, where mating induced transcriptomic changes in the female mosquito RT have been reported [36, 38], and at 72 h, when eggs are fully developed [62].

## Results

We examined microbial profiles of lab-reared *Ae. aegypti* and *Ae. albopictus*, identifying the bacterial microbiota of three tissues: the gut, URT (females: ovaries; males: testes), and LRT (females: oviduct, spermathecae, spermathecal vestibule, and bursa; males: vas deferens, accessory glands, and seminal vesicles). We determined bacterial composition in virgins of both sexes, and in mated females at 0, 24, 48, and 72 h post-mating; 72 h is the typical time a female requires to lay a clutch of eggs after blood ingestion in the strains used in our experiments. Virgin females were also examined at each time point as an age control. Mated females were further split into two groups: mated only and mated + blood-fed (a blood meal was given to a subset of females shortly after mating, see “Methods”); these female groups are referred to hereafter as non-blood-fed (NBF) and blood-fed (BF). We also identified bacteria in several control samples (whole adults, larval rearing water, sucrose solution, fish food pellets, 1× PBS, and DNA extraction reagents; Additional file 1: Table S1).

**Fig. 1:**
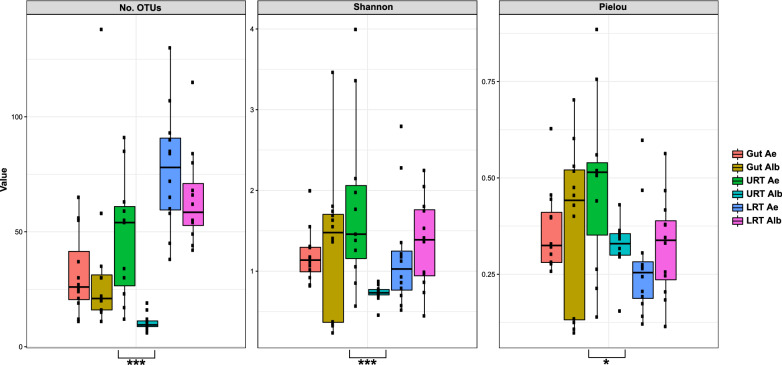
Alpha diversity indicators, **a** Number of OTUs, **b** Shannon diversity index, and **c** Pielou’s evenness index for *Ae. aegypti* (Ae) and *Ae. albopictus* (Alb) tissue samples. Asterisks indicate statically significant differences between species. *P = 0.05–0.005, **P = 0.0049–0.0005, ***P < 0.00049

Overall, 72 tissue and 19 control samples were sequenced (Additional file 1: Table S1), identifying a total of 1,536,017 sequences and 2493 OTUs. After OTU clustering, one tissue sample (*Ae. aegypti* virgin 24 h URT) was discarded as the low-abundance OTU overestimation evidenced a highly contaminated sample. The final tissue samples contained an average of 16,219 ± 7905 sequences and 44 ± 31 OTUs. The sampling has a complete coverage according to Good’s index (Additional file 1: Table S1), with most of the samples reaching the plateau in rarefaction curves with a subsampling of 5000 sequences (Additional file 2: Figure S1). For alpha diversity (Fig. 1), richness (number of OTUs) was higher for LRT samples, while the diversity (Shannon index) and evenness (Pielou’s index) were similar between all tissues, excluding the *Ae. albopictus* URT, which had the lowest richness and diversity of all tissues examined. The URT was the only tissue with a statistically significant difference between *Ae. aegypti* and *Ae. albopictus* for all estimators (number of OTUs, *W* = 128.5, DF = 1,  *P* = 0.0001; Shannon, *W* = 120, DF = 1,  *P* = 0.0004; Pielou’s, *W* = 98, DF = 1, *P* = 0.024).

### Community composition in tissue and control samples

**Fig. 2:**
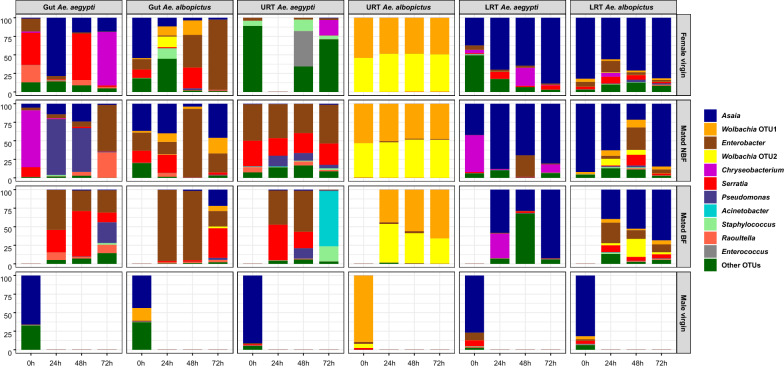
Relative OTU abundance in gut, URT, and LRT tissues from virgin females, mated NBF females, mated BF females, and virgin males at the specified time points. The 11 most abundant OTUs are shown, with those outside this group represented in the “other OTUs” category

Eleven OTUs had an overall abundance of ≥ 1% (Fig. 2; abundance is shown in Additional file 3: Table S2). OTU distribution varied according to the host species, sex, sampled tissue, and treatment. For males, we only sampled virgin individuals of the same age, finding that the bacterial community shared the same dominant OTUs. The most common bacteria for both *Ae. aegypti* and *Ae. albopictus* across the different male tissues was *Asaia.* In male *Ae. aegypti*, the average relative abundance (ARA) of *Asaia* in the gut, URT, and LRT was 65.87%, 91.11%, and 76.82%, respectively. For male *Ae. albopictus*, the *Asaia* ARA for the gut and LRT was 43.64% and 81.64%, respectively. The *Ae. albopictus* URT was the only male tissue with a distinct microbiota, dominant for a *Wolbachia* OTU (89.38% ARA).

In females, gut tissue displayed the most differences between treatments: in virgin *Ae. aegypti*, the dominant OTUs were *Asaia* and *Serratia* (29.45% and 27.25% ARA, respectively). *Pseudomonas* was also dominant in mated NBF females, (33.86% ARA), with a high relative abundance of 28% in one BF sample (72 h). *Enterobacter* and *Serratia* were dominant in BF females (37.15% and 34.86% ARA, respectively). *Chryseobacterium* was the dominant bacteria in one virgin (72 h) and one mated NBF (0 h) sample, with a relative abundance ˃ 70% in both cases. In the *Ae. albopictus* gut, *Enterobacter* was the most abundant OTU for all three female groups, virgin (38.45% ARA), mated NBF (39.67% ARA ), and mated BF females (69.95% ARA). *Asaia* was also abundant in NBF *Ae. albopictus* females (31.55% ARA).

The *Ae. aegypti* female URT tissue had a similar composition to the gut tissue, sharing some of the abundant OTUs. Except for virgin samples, where no abundant OTUs were dominant. *Enterobacter* was the most abundant OTU in mated NBF females (46.56% ARA) and BF females (34.12% ARA). Other abundant OTUs were *Serratia* in mated NBF females (28.38% ARA) and BF females (23.47% ARA), and *Acinetobacter*, the dominant bacteria in the 72 h BF sample, with a relative abundance of 75%. The *Ae. albopictus* URT had a unique community compared to other tissues of this species and compared to *Ae. aegypti*, with two *Wolbachia* OTUs dominant in virgin and mated females (ARA ≥ 97%); the presence of both *Wolbachia* strains in our laboratory colony was confirmed by PCR using diagnostic wsp primers [63] for *Wolbachia* supergroup A and B (Additional file 4: Figure S2).

The LRT was the only tissue where females of both species had a similar community structure, with *Asaia* being the dominant OTU for all treatments, with ARA greater than 50%. *Asaia* ARA for virgin, NBF, and BF females was 65.04%, 70.04%, and 59.74% in the *Ae. aegypti* LRT, and 72.63%, 65.21%, and 53.43% in the *Ae. albopictus* LRT. Other abundant OTUs for *Ae. aegypti* were *Chryseobacterium*, which reached abundance between 11 and 50% in some of the virgin, BF, and NBF LRT samples, and *Enterobacter*, with relative abundance of 29% in the mated NBF sample at 48 h. This same *Enterobacter* OTU was also found in *Ae. albopictus* samples in abundance ranging from 10 to 30% at the three post-mating time points. Further, the abundance of the two *Wolbachia* OTUs was lower in the LRT than in the URT, with only one sample (48 h BF) reaching 24%.

The distribution of the most abundant OTUs was also analyzed in the control samples (Additional file 3: Table S2 and Additional file 5: Figure S3). In female whole bodies, *Raoultella*, *Serratia*, and *Enterobacter* were dominant in *Ae. aegypti* (27.55%, 20.15%, and 10.83% ARA, respectively), while *Enterobacter* was highly dominant in *Ae. albopictus* (89.84% ARA). In male whole-bodies for both species, *Asaia* was the most abundant OTU (ARA of 23.72% in *Ae. aegypti*, and 36.91% in *Ae. albopictus*). In the larval rearing water, sucrose solution, and fish food samples, the dominant OTUs in tissue samples were mostly found in low abundance, except for *Asaia*, which had significant abundance in the final sucrose solution samples (ARA of 36.76% in *Ae. aegypti*, and 26.31% in *Ae. albopictus*).

### Community assemblage analyses

The indicator species analysis was performed using the subsampling of OTUs with a total overall abundance ≥ 0.001% (281 OTUs in total) with a focus on results with high indicator values (≥ 0.75; Table 1). At the host species level, the only OTUs with significant results were two *Wolbachia* OTUs associated with *Ae. albopictus*. For the other evaluated variables (tissue, reproductive status, and nutritional status), we performed the analyses separately for each species to increase its sensitivity. The most significant variable was the host tissue: *Raoultella* was associated with gut tissue of both *Ae. aegypti* and *Ae. albopictus*, while *Enterobacter* and *Serratia* were associated with only the *Ae. albopictus* gut. In the URT, *Staphylococcus* was associated with *Ae. aegypti* samples. The *Ae. aegypti* LRT was the tissue with the most associated OTUs, 10 in total. At the reproductive level (i.e., mating status), only two OTUs, *Chryseobacterium* and *Pseudomonas*, were associated with virgin and mated *Ae. aegypti*, respectively. Finally, for nutritional status, no bacteria were associated with blood-feeding or non-blood-feeding (i.e., exclusively sucrose).

**Table 1:** Indicator species analysis for OTUs with an overall abundance ≥ 0.001%

	OTU code	Classification	Indicator value	***P***-value	Associated variable
Species level	Otu0003	*Wolbachia*	0.97	0.001	*Ae. albopictus*
	Otu0004	*Wolbachia*	0.91	0.001	*Ae. albopictus*
Tissue level	Otu00010	*Raoultella*	0.94	0.001	Gut—*Ae. aegypti*
	Otu00009	*Staphylococcus*	0.94	0.001	URT—*Ae. aegypti*
	Otu00020	Microbacteriaceae unclassified	0.99	0.001	LRT—*Ae. aegypti*
	Otu00014	*Delftia*	0.97	0.001	LRT—*Ae. aegypti*
	Otu00080	*Nocardioides*	0.95	0.001	LRT—*Ae. aegypti*
	Otu00024	Alphaproteobacteria unclassified	0.87	0.001	LRT—*Ae. aegypti*
	Otu00026	*Arthrobacter*	0.86	0.001	LRT—*Ae. aegypti*
	Otu00001	*Asaia*	0.85	0.001	LRT—*Ae. aegypti*
	Otu00029	*Acinetobacter*	0.84	0.001	LRT—*Ae. aegypti*
	Otu00019	Sphingobacteriaceae unclassified	0.81	0.001	LRT—*Ae. aegypti*
	Otu00015	*Sphingobacterium*	0.80	0.002	LRT—*Ae. aegypti*
	Otu00060	Chitinophagaceae unclassified	0.77	0.001	LRT—*Ae. aegypti*
	Otu00002	*Enterobacter*	0.94	0.001	Gut—*Ae. albopictus*
	Otu00005	*Serratia*	0.79	0.003	Gut—*Ae. albopictus*
	Otu00010	*Raoultella*	0.79	0.001	Gut—*Ae. albopictus*
Reproductive level	Otu00021	*Chryseobacterium*	0.79	0.001	Virgin—*Ae. aegypti*
	Otu00008	*Pseudomonas*	0.90	0.001	Mated—*Ae. aegypti*

Only OTUs with a high indicator value (≥ 0.75) and a *P*-value ≤ 0.05 are shown. Associated variables evaluated are host species and tissue, reproductive, and nutritional status for each species

**Fig. 3:**
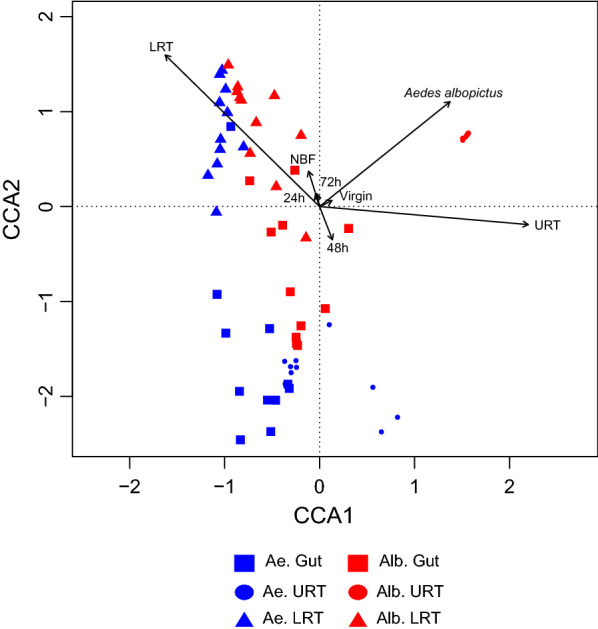
CCA plot of bacterial community structure in the mosquito tissue samples. Independent variables are host species, tissue, time point, reproductive and nutritional status. Arrow length indicates the strength of correlation between variables and ordination scores

The PERMANOVA analysis for host species, sampled tissues, time point, mating status, and nutritional status variables shows that tissue (*R*^2^ = 0.29; *P* = 0.01), and host species (*R*^2^ = 0.08; *P* = 0.01) better explain the bacterial community composition, compared to the time point (*R*^2^ = 0.02; *P* = 0.83), reproductive status (*R*^2^ = 0.01; *P* = 0.27), and nutritional status (*R*^2^ = 0.009; *P* = 0.52). This result is confirmed by the CCA (Fig. 3), where clusters defined by host species and tissue can be observed. This is most notable in *Ae. albopictus* URT samples, which form an isolated cluster distinguishable from the other sample types due to the dominance of *Wolbachia* (Fig. 3).

### Predicted bacterial metabolic pathways

We used PICRUSt 2.0 to analyze the predicted bacterial functional profile for the tissue samples. Based on the OTU taxonomy and relative abundance in each sample, we predicted bacterial metabolic pathways to determine the effect of mating and blood-feeding on microbial physiology. This analysis predicted 420 predicted metabolic pathways overall. We focused on abundance differential testing only in the URT and LRT at two time points: at 24 h, when large-scale mating induced transcriptomic changes occur [36, 38], and at 72 h, when eggs are fully developed after blood-feeding [62]. The comparisons were made between (1) virgin vs. mated NBF females, and (2) mated NBF vs. mated BF females. Because the *Ae. aegypti* 24 h virgin URT sample was discarded, we used the 48 h vs. 72 h for this comparison. Most of the resulting pathways belong to three superclasses according to the classification of the Metacyc database: “Biosynthesis,” “Degradation/Utilization/Assimilation,” and “Generation of Precursor Metabolites and Energy.”

In total, 16 comparison groups were identified. The number of pathways with abundance differences ranged from only two (*Ae. albopictus* BF vs. NBF URT at 24 h) to 92 (*Ae. aegypti* BF vs. NBF URT at 72 h) (Additional file 6: Table S3). More overabundance pathways were found in the URT compared to the LRT. In the virgin vs. NBF group, we found 225 pathways in the URT compared to 86 in the LRT. In the NBF vs. BF group, 186 pathways were found in the URT compared to 84 in LRT. At the species level, for most of the comparison groups, more overabundant pathways were found in *Ae. aegypti* microbiota compared to *Ae. albopictus* communities, except for the virgin vs. NBF LRT at 72 h and NBF vs. BF LRT at 24 h.

Finally, we examined overabundant pathways shared between both host species microbiota (Additional file 7: Table S4). In general, few pathways were shared, with two cases having no shared pathways (NBF vs. BF URT at 24 h, and NBF vs. BF LRT at 72 h). The groups with more identified pathways were the virgin vs. NBF URT at 24 h and NBF vs. mated BF URT at 72 h, with 21 and 26 shared pathways, respectively. For the temporal comparison, we analyzed which overabundant pathways were shared at 24 and 72 h for each host species (Additional file 8: Table S5). Like the species comparison, very few pathways were shared, with none in the *Ae. albopictus* NBF vs. BF URT. As expected, more shared pathways were found in the *Ae. aegypti* virgin vs. NBF URT, as the comparison was made between 48 and 72 h samples.

## Discussion

### Bacterial diversity in *Aedes aegypti* and *Aedes albopictus* tissues

We characterized the microbiota of the gut, URT, and LRT of *Ae. aegypti* and *Ae. albopictus*. The predominant OTUs belonged to three phyla, Proteobacteria, Bacteroides, and Firmicutes, consistent with previous reports of mosquito-associated communities [27]. At the genus level, the taxonomic composition is vastly more diverse. This variability is proposed to be conditioned by environmental factors (i.e., breeding site and food source) rather than by host physiological factors [25, 26, 28]. Our results also evidence that the primary source of bacterial diversity is the sampled tissue, indicating a relevant bacterial tropism previously reported for mosquito tissues [30, 31]. Most OTUs identified in the examined tissues were also found in the larval rearing water and in sucrose fed to adults, indicating an environmental transmission route common for mosquito microbiota [26]. The most abundant OTUs belonged to Gammaproteobacteria previously described as part of the *Aedes* gut microbiota: *Acinetobacter* [64, 65], *Enterobacter* [64, 66, 67], *Pseudomonas* [64, 66, 68]*,* and *Serratia* [31, 69]*. Acinetobacter* and *Pseudomonas* have also been described in *Ae. aegypti* male and female gonads [31]. The vertically transmitted Alphaproteobacteria *Wolbachia* was also abundant in the *Ae. albopictus* URT, with OTUs corresponding to the wAlbA and wAlbB strains [70].

The most abundant overall OTU in our sampling was the Alphaproteobacteria *Asaia,* a member of the acetic acid bacteria group associated with sugar-fed insects [71]. *Asaia* was dominant in the LRTs of both species but absent in their respective URTs. *Wolbachia* was dominant in the *Ae. albopictus* URT and reciprocal negative interference between *Wolbachia* and *Asaia* has been proposed [72]. *Asaia* was originally described in *Anopheles* [73], associated with larvae and adult survival [74–76], and later in *Ae. aegypti* and *Ae. albopictus* gut tissue [31, 64, 72] and *Ae. aegypti* female and male gonads [31], with no association with *Aedes* physiology yet described. *Asaia* has multiple routes of transmission: nectar feeding [24], sexual transmission [77], and vertical transmission with its presence on the egg surface suggesting maternal transmission via egg-smearing [72, 78, 79]. We cannot propose a vertical transmission of *Asaia* in our assays due to its absence in the ovary and the lack of sampling of mosquito eggs. Although the abundance of *Asaia* in sucrose suggests transmission by feeding, *Asaia* was only found in sucrose from adult cages 4–6 d after mosquito release, suggesting other routes of transmission such as excretion in feces or inoculation via saliva, as *Asaia* has been described in salivary glands in other mosquito species [31].

### Microbiota and host physiology

Insect seminal fluid often contains antimicrobial peptides that are transferred to females during mating [80, 81]. Mating also stimulates expression of genes with antibacterial properties [82]. *Aedes aegypti* and *Ae. albopictus* seminal fluid also contains immunity-related proteins [33–35], and mating induces the expression of genes with antimicrobial functions in the female RT [36–38]. Female mosquitoes also require a blood meal for egg development, with blood ingestion altering microbiota composition in the mosquito gut [41]. Further, mating and blood-feeding regulate the expression of genes with roles in oxidative stress in the *Ae. aegypti* female RT [36, 38]. Therefore, we examined the microbiota composition in each tissue from virgin and mated females (NBF), and in mated females subsequently blood-fed (BF) in both *Ae. aegypti* and *Ae. albopictus*.

PERMANOVA and indicator species analysis do not evidence a statistically significant effect of diet on the microbiota taxonomic structure. Instead, we found overall relative abundance changes in the gut microbiota of OTUs associated with specific diet treatments. *Serratia* and *Enterobacter*, bacteria with strong hemolytic activity and associated with blood digestion in *Ae. aegypti* [44, 83], had larger relative abundances in BF females than in virgin or NBF females of both species. Recently, *Enterobacteriaceae* was shown to interfere with *Serratia* colonization in the *Ae. aegypti* gut [45], and the presence of *Serratia* interfered with feeding activity [45], showing that microbial interactions can influence adult behavior in this species.

No significant differences in the microbiota structure were observed between virgin and mated females; only two OTUs in *Ae. aegypti* had a higher abundance associated with *Ae. aegypti* mating status (*Chryseobacterium* in virgin and *Pseudomonas* in mated samples, respectively). As our study was qualitative and not quantitative, we could not establish changes in the microbial load in the LRT between virgin and mated insects. Blood-feeding induces egg development [84, 85] and ovary growth. In *Ae. albopictus*, independent of mating status and egg development stage, *Wolbachia* was dominant, showing its ability to colonize this tissue over other groups [72, 86]. *Aedes aegypti* showed an increase in *Acinetobacter* and *Staphylococcus* in fully developed ovary tissue at 72 h compared to virgin and NBF females. *Acinetobacter* has been previously described as a common ovary resident in lab-reared *Ae. aegypti* [31, 32].

When we examined the predicted metabolic profiles of the tissue-associated microbiota, we observed differences between groups. In NBF females of both species at 24 h, there was an overabundance in microbial energy production pathways in the URT, including aerobic respiration and fermentation pathways. Sugar fermentation is common in gut microbiota of insects with a sugar-rich diet, such as bees [87, 88]. We also observed an increase *Enterobacter* and *Serratia* abundance in NBF female ovaries. *Enterobacterales* are facultative anaerobes that can switch to sugar fermentation under conditions of oxygen depletion [89]. In NBF and BF samples, most shared overabundance pathways were present at 72 h. The pathways found included the following: (1) Pathways related to sugar degradation. (2) Pathways exclusive to *Enterobacteriaceae*, synthesis of enterobacterial common antigen (ECA), an outer membrane glycolipid antigen [90], and enterobactin synthesis, a siderophore. Siderophores are highly represented in the biosynthetic gene clusters of *An. gambiae* microbiota [91]. (3) Quinone synthesis pathways, molecules part of the electron transport chain with additional roles in Gram-positive bacteria sporulation [92, 93]. The predicted functional profiles do have two limitations: (1) accuracy depends on existing reference genomes, and (2) some bacteria have similar 16S rRNA hypervariable regions despite being genotypically divergent [59, 94]. We expect that further in silico and metatranscriptomics studies will lead to a better understanding of the functional role of the mosquito microbiota.

## Conclusions

Our comparative analysis shows that *Ae. aegypti* and *Ae. albopictus* have unique RT microbiota, with the dominant abundance of *Wolbachia* in *Ae. albopictus* gonads being the most notable difference between the species. Also, no major differences were observed in virgin, mated, or blood-fed females of either species. Some of the bacteria identified here, such as *Asaia* and *Wolbachia*, have tissue-specific tropism for the RT, further supporting their potential use in vector control strategies. On the other hand, the predicted functional profiles of RT-associated microbiota show changes in the use of pathways associated with these events, suggesting a physiological interaction between microbiota and host that could influence mosquito reproduction and/or other physiological processes. The influence of RT tissue microbiota requires further elucidation using axenic or gnotobiotic models [95] to further evaluate the role of the microbiota on the reproductive behaviors and fertility of these vector species.

## Supplementary Information

**Additional file 1: Table S1.** Tissue and control sample description, including relevant metadata and alpha-diversity estimators.

**Additional file 2: Figure S1.** Rarefaction curves of tissue samples per host species and controls.

**Additional file 3: Table S2.** Relative abundance for the most abundant OTUs. The 11 most abundant OTUs are shown, with those outside this group represented in the “other OTUs” category. In black, the average abundance for each sample type at all time points.

**Additional file 4: Figure S2.** Agarose gel for three whole-body female *Ae. albopictus* from the Medellín strain laboratory colony. Lanes 1–3, individual samples; lane 4, negative control (NC); lane 5, molecular weight size marker. Band of 501 bp corresponds to wAlbB (*Wolbachia* supergroup B) and band of 379 bp to wAlbA (*Wolbachia* supergroup A).

**Additional file 5: Figure S3.** Relative OTUs abundance for control samples, virgin whole-body insects, larval rearing water, sucrose solution, food pellet, 1× PBS, and DNA extraction reagents. The 11 most abundant OTUs are shown, with those outside this group represented in the “other OTUs” category.

**Additional file 6: Table S3.** Predicted metabolic pathways with a significant change in their abundance according to the comparison group (virgin vs. mated; mated NBF vs. mated BF). Summary of the results in the first sheet with the results for each group organized according to their code.

**Additional file 7: Table S4.** Shared pathways with significant abundance changes between *Ae. aegypti* and *Ae. albopictus* according to the comparison group. The number of shared pathways is shown in parenthesis.

**Additional file 8: Table S5.** Shared pathways with significant abundance change between 24 and 72 h time points according to the comparison group. The number of shared pathways is shown in parenthesis.

## Data Availability

The datasets generated and analyzed during the current study are available in the NCBI Sequence Read Archive (SRA) repository, under the BioProject with accession code PRJNA644640 (http://www.ncbi.nlm.nih.gov/bioproject/644640).

## Abbreviations

CPM: Counts per million
URT: Upper reproductive tract
LRT: Lower reproductive tract
STE: Sodium chloride-Tris–EDTA
OTU: Operational taxonomic unit
ASV: Amplicon sequence variant
DCA: Detrended canonical correspondence analysis
CCA: Canonical correspondence analysis
BVC: Biological coefficient of variation
NBF: Non-blood-fed
BF: Blood-fed
ARA: Average relative abundance

## References

[1] Brady OJ, Gething PW, Bhatt S, Messina JP, Brownstein JS, Hoen AG (2012). Refining the global spatial limits of dengue virus transmission by evidence-based consensus. PLoS Negl Trop Dis..

[2] Alfonso-Parra C, Avila FW (2018). Molecular responses to the Zika virus in mosquitoes. Pathogens.

[3] Lounibos LP, Kramer LD (2016). Invasiveness of *Aedes aegypti* and *Aedes albopictus* and vectorial capacity for chikungunya virus. J Infect Dis.

[4] Kraemer MUG, Sinka ME, Duda KA, Mylne AQN, Shearer FM, Barker CM (2015). The global distribution of the arbovirus vectors *Aedes aegypti* and *Ae. albopictus*. Elife..

[5] Poelchau MF, Reynolds JA, Elsik CG, Denlinger DL, Armbruster PA (2013). Deep sequencing reveals complex mechanisms of diapause preparation in the invasive mosquito, *Aedes albopictus*. Proc R Soc B Biol Sci.

[6] Marini G, Manica M, Arnoldi D, Inama E, Rosà R, Rizzoli A (2020). Influence of temperature on the life-cycle dynamics of *Aedes albopictus* population established at temperate latitudes: a laboratory experiment. Insects.

[7] Medley KA, Westby KM, Jenkins DG (2019). Rapid local adaptation to northern winters in the invasive Asian tiger mosquito *Aedes albopictus:* a moving target. J Appl Ecol.

[8] Kraemer MUG, Reiner RC, Brady OJ, Messina JP, Gilbert M, Pigott DM (2019). Past and future spread of the arbovirus vectors *Aedes aegypti* and *Aedes albopictus*. Nat Microbiol.

[9] Hopkins BR, Avila FW, Wolfner MF (2018). Insect male reproductive glands and their products. Encycl Reprod.

[10] Avila FW, Sirot LK, LaFlamme BA, Rubinstein CD, Wolfner MF (2011). Insect seminal fluid proteins: identification and function. Annu Rev Entomol.

[11] Schnakenberg SL, Matias WR, Siegal ML (2011). Sperm-storage defects and live birth in drosophila females lacking spermathecal secretory cells. PLoS Biol..

[12] Yan J, Kibech R, Stone CM (2021). Differential effects of larval and adult nutrition on female survival, fecundity, and size of the yellow fever mosquito *Aedes aegypti*. Front Zool.

[13] Telang A, Wells MA (2004). The effect of larval and adult nutrition on successful autogenous egg production by a mosquito. J Insect Physiol.

[14] Schwenke RA, Lazzaro BP, Wolfner MF (2016). Reproduction–immunity trade-offs in insects. Annu Rev Entomol.

[15] Agudelo J, Alfonso-Parra C, Avila FW (2021). Male age influences re-mating incidence and sperm use in females of the dengue vector *Aedes aegypti*. Front Physiol.

[16] Helinski MEH, Harrington LC (2011). Male mating history and body size influence female fecundity and longevity of the dengue vector *Aedes aegypti*. J Med Entomol.

[17] Ramírez-Sánchez LF, Camargo C, Avila FW (2020). Male sexual history influences female fertility and re-mating incidence in the mosquito vector *Aedes aegypti* (Diptera: Culicidae). J Insect Physiol..

[18] Carrington LB, Armijos MV, Lambrechts L, Barker CM, Scott TW (2013). Effects of fluctuating daily temperatures at critical thermal extremes on *Aedes aegypti* life-history traits. PLoS ONE..

[19] Gould AL, Zhang V, Lamberti L, Jones EW, Obadia B, Korasidis N (2018). Microbiome interactions shape host fitness. Proc Natl Acad Sci.

[20] Morimoto J, Simpson SJ, Ponton F (2017). Direct and trans-generational effects of male and female gut microbiota in *Drosophila melanogaster*. Biol Lett.

[21] Delbare SYN, Ahmed-Braimah YH, Wolfner MF, Clark AG (2020). Interactions between the microbiome and mating influence the female’s transcriptional profile in *Drosophila melanogaster*. Sci Rep.

[22] Gimonneau G, Tchioffo MT, Abate L, Boissière A, Awono-Ambene PH, Nsango SE (2014). Composition of *Anopheles coluzzii* and *Anopheles gambiae* microbiota from larval to adult stages. Infect Genet Evol.

[23] Coon KL, Brown MR, Strand MR (2016). Mosquitoes host communities of bacteria that are essential for development but vary greatly between local habitats. Mol Ecol.

[24] Hubert B, Amadou NEH, Florence F, Souleymane D, Ousmane F, Didier R (2020). Role of plants in the transmission of *Asaia* sp., which potentially inhibit the Plasmodium sporogenic cycle in Anopheles mosquitoes. Sci Rep.

[25] Strand MR (2017). Chapter 11 The gut microbiota of mosquitoes diversity and function. Arthropod vector: controller of disease transmission.

[26] Strand MR (2018). Composition and functional roles of the gut microbiota in mosquitoes. Curr Opin Insect Sci.

[27] Scolari F, Casiraghi M, Bonizzoni M (2019). *Aedes* spp. and their microbiota: a review. Front Microbiol..

[28] Guégan M, Zouache K, Démichel C, Minard G, Potier P, Mavingui P (2018). The mosquito holobiont: fresh insight into mosquito-microbiota interactions. Microbiome.

[29] Pike A, Dong Y, Dizaji NB, Gacita A, Mongodin EF, Dimopoulos G (2017). Changes in the microbiota cause genetically modified *Anopheles* to spread in a population. Science.

[30] Segata N, Baldini F, Pompon J, Garrett WS, Truong DT, Dabiré RK (2016). The reproductive tracts of two malaria vectors are populated by a core microbiome and by gender-and swarm-enriched microbial biomarkers. Sci Rep.

[31] Mancini MV, Damiani C, Accoti A, Tallarita M, Nunzi E, Cappelli A (2018). Estimating bacteria diversity in different organs of nine species of mosquito by next generation sequencing. BMC Microbiol.

[32] Alvarado WA, Agudelo SO, Velez ID, Vivero RJ (2021). Description of the ovarian microbiota of *Aedes aegypti* (L.) Rockefeller strain. Acta Trop..

[33] Boes KE, Ribeiro JMC, Wong A, Harrington LC, Wolfner MF, Sirot LK (2014). Identification and characterization of seminal fluid proteins in the Asian tiger mosquito, *Aedes albopictus*. PLoS Negl Trop Dis..

[34] Sirot LK, Poulson RL, McKenna MC, Girnary H, Wolfner MF, Harrington LC (2008). Identity and transfer of male reproductive gland proteins of the dengue vector mosquito, *Aedes aegypti*: potential tools for control of female feeding and reproduction. Insect Biochem Mol Biol.

[35] Degner EC, Ahmed-Braimah YH, Borziak K, Wolfner MF, Harrington LC, Dorus S (2018). Proteins, transcripts, and genetic architecture of seminal fluid and sperm in the mosquito *Aedes aegypti*. Mol Cell Proteomics.

[36] Alfonso-Parra C, Ahmed-Braimah YH, Degner EC, Avila FW, Villarreal SM, Pleiss JA (2016). Mating-induced transcriptome changes in the reproductive tract of female *Aedes aegypti*. PLoS Negl Trop Dis..

[37] Pascini TV, Ramalho-Ortigão M, Ribeiro JM, Jacobs-Lorena M, Martins GF (2020). Transcriptional profiling and physiological roles of *Aedes aegypti* spermathecal-related genes. BMC Genomics.

[38] Camargo C, Ahmed-Braimah YH, Amaro IA, Harrington LC, Wolfner MF, Avila FW (2020). Mating and blood-feeding induce transcriptome changes in the spermathecae of the yellow fever mosquito *Aedes aegypti*. Sci Rep.

[39] Toh SQ, Glanfield A, Gobert GN, Jones MK (2010). Heme and blood-feeding parasites: friends or foes?. Parasit Vectors.

[40] Wang Y, Gilbreath TM, Kukutla P, Yan G, Xu J (2011). Dynamic gut microbiome across life history of the malaria mosquito *Anopheles gambiae* in Kenya. PLoS ONE..

[41] Oliveira JHM, Gonçalves RLS, Lara FA, Dias FA, Gandara ACP, Menna-Barreto RFS (2011). Blood meal-derived heme decreases ROS levels in the midgut of *Aedes aegypti* and allows proliferation of intestinal microbiota. PLoS Pathog..

[42] Engl T, Kaltenpoth M (2018). Influence of microbial symbionts on insect pheromones. Nat Prod Rep.

[43] Coon KL, Brown MR, Strand MR (2016). Gut bacteria differentially affect egg production in the anautogenous mosquito Aedes aegypti and facultatively autogenous mosquito *Aedes atropalpus* (Diptera: Culicidae). Parasit Vectors.

[44] de Gaio A, Gusmão DS, Santos AV, Berbert-Molina MA, Pimenta PFP, Lemos FJA (2011). Contribution of midgut bacteria to blood digestion and egg production in Aedes aegypti (Diptera: Culicidae) (L.). Parasit Vectors..

[45] Kozlova EV, Hegde S, Roundy CM, Golovko G, Saldaña MA, Hart CE (2021). Microbial interactions in the mosquito gut determine *Serratia* colonization and blood-feeding propensity. ISME J.

[46] Hughes GL, Dodson BL, Johnson RM, Murdock CC, Tsujimoto H, Suzuki Y (2014). Native microbiome impedes vertical transmission of *Wolbachia* in *Anopheles* mosquitoes. Proc Natl Acad Sci.

[47] Dennison NJ, Saraiva RG, Cirimotich CM, Mlambo G, Mongodin EF, Dimopoulos G (2016). Functional genomic analyses of *Enterobacter, Anopheles* and *Plasmodium* reciprocal interactions that impact vector competence. Malar J.

[48] Degner EC, Harrington LC (2016). Polyandry depends on postmating time interval in the dengue vector *Aedes aegypti*. Am J Trop Med Hyg.

[49] Oliva CF, Damiens D, Vreysen MJB, Lemperière G, Gilles J (2013). Reproductive strategies of *Aedes albopictus* (Diptera: Culicidae) and implications for the sterile insect technique. PLoS ONE..

[50] Herlemann DP, Labrenz M, Jürgens K, Bertilsson S, Waniek JJ, Andersson AF (2011). Transitions in bacterial communities along the 2000 km salinity gradient of the Baltic Sea. ISME J.

[51] Schloss PD, Westcott SL, Ryabin T, Hall JR, Hartmann M, Hollister EB (2009). Introducing mothur: open-source, platform-independent, community-supported software for describing and comparing microbial communities. Appl Environ Microbiol.

[52] Rognes T, Flouri T, Nichols B, Quince C, Mahé F (2016). VSEARCH: a versatile open source tool for metagenomics. Peer J..

[53] Pruesse E, Oliver F, Wren J (2012). SINA: accurate high throughput multiple sequence alignment of ribosomal RNA genes. Bioinformatics.

[54] Díaz S, Escobar JS, Avila FW (2021). Identification and removal of potential contaminants in 16S rRNA gene sequence data sets from low-microbial-biomass samples: an example from mosquito tissues. Msphere.

[55] Dufrêne M, Legendre P (1997). Species assemblages and indicator species: the need for a flexible asymmetrical approach. Ecol Monogr.

[56] Roberts DW, Roberts MDW, Package ‘labdsv’. In: Ordination and multivariate. 2016.

[57] Anderson MJ (2001). A new method for non-parametric multivariate analysis of variance. Austral Ecol.

[58] Dixon P (2003). VEGAN, a package of R functions for community ecology. J Veg Sci.

[59] Douglas GM, Maffei VJ, Zaneveld JR, Yurgel SN, Brown JR, Taylor CM (2020). PICRUSt2 for prediction of metagenome functions. Nat Biotechnol.

[60] Caspi R, Billington R, Keseler IM, Kothari A, Krummenacker M, Midford PE (2020). The MetaCyc database of metabolic pathways and enzymes-a 2019 update. Nucleic Acids Res.

[61] Risso D, Ngai J, Speed TP, Dudoit S (2014). Normalization of RNA-seq data using factor analysis of control genes or samples. Nat Biotechnol.

[62] Robinson MD, McCarthy DJ, Smyth GK (2010). edgeR: a Bioconductor package for differential expression analysis of digital gene expression data. Bioinformatics.

[63] Zhou W, Rousset F, O’Neill S (1998). Phylogeny and PCR–based classification of *Wolbachia* strains using wsp gene sequences. Proc R Soc London Ser B Biol Sci.

[64] David MR, dos Santos LMB, Vicente ACP, Maciel-de-Freitas R (2016). Effects of environment, dietary regime and ageing on the dengue vector microbiota: evidence of a core microbiota throughout Aedes aegypti lifespan. Mem Inst Oswaldo Cruz.

[65] Minard G, Tran F-H, Goubert C, Bellet C, Lambert G, Khanh HKL (2015). French invasive Asian tiger mosquito populations harbor reduced bacterial microbiota and genetic diversity compared to Vietnamese autochthonous relatives. Front Microbiol..

[66] Dickson LB, Ghozlane A, Volant S, Bouchier C, Ma L, Vega-Rúa A (2018). Diverse laboratory colonies of *Aedes aegypti* harbor the same adult midgut bacterial microbiome. Parasit Vectors..

[67] Dickson LB, Jiolle D, Minard G, Moltini-Conclois I, Volant S, Ghozlane A (2017). Carryover effects of larval exposure to different environmental bacteria drive adult trait variation in a mosquito vector. Sci Adv..

[68] Rosso F, Tagliapietra V, Albanese D, Pindo M, Baldacchino F, Arnoldi D (2018). Reduced diversity of gut microbiota in two *Aedes* mosquitoes species in areas of recent invasion. Sci Rep.

[69] Muturi EJ, Dunlap C, Ramirez JL, Rooney AP, Kim C-H (2019). Host blood-meal source has a strong impact on gut microbiota of *Aedes aegypti*. FEMS Microbiol Ecol..

[70] Sinkins SP, Braig HR, O’Neill SL (1995). *Wolbachia* superinfections and the expression of cytoplasmic incompatibility. Proc R Soc London Ser B Biol Sci.

[71] Crotti E, Rizzi A, Chouaia B, Ricci I, Favia G, Alma A (2010). Acetic acid bacteria, newly emerging symbionts of insects. Appl Environ Microbiol.

[72] Rossi P, Ricci I, Cappelli A, Damiani C, Ulissi U, Mancini MV (2015). Mutual exclusion of *Asaia* and *Wolbachia* in the reproductive organs of mosquito vectors. Parasit Vectors.

[73] Favia G, Ricci I, Damiani C, Raddadi N, Crotti E, Marzorati M (2007). Bacteria of the genus *Asaia* stably associate with *Anopheles stephensi*, an Asian malarial mosquito vector. Proc Natl Acad Sci.

[74] Chouaia B, Rossi P, Epis S, Mosca M, Ricci I, Damiani C (2012). Delayed larval development in *Anopheles* mosquitoes deprived of *Asaia* bacterial symbionts. BMC Microbiol.

[75] Mitraka E, Stathopoulos S, Siden-Kiamos I, Christophides GK, Louis C (2013). *Asaia* accelerates larval development of *Anopheles gambiae*. Pathog Glob Health.

[76] Mancini MV, Damiani C, Short SM, Cappelli A, Ulissi U, Capone A (2020). Inhibition of *Asaia* in adult mosquitoes causes male-specific mortality and diverse transcriptome changes. Pathogens.

[77] Damiani C, Ricci I, Crotti E, Rossi P, Rizzi A, Scuppa P (2008). Paternal transmission of symbiotic bacteria in malaria vectors. Curr Biol..

[78] Damiani C, Ricci I, Crotti E, Rossi P, Rizzi A, Scuppa P (2010). Mosquito-bacteria symbiosis: the case of *Anopheles gambiae* and *Asaia*. Microb Ecol..

[79] Mancini MV, Spaccapelo R, Damiani C, Accoti A, Tallarita M, Petraglia E (2016). Paratransgenesis to control malaria vectors: a semi-field pilot study. Parasit Vectors.

[80] Lung O, Kuo L, Wolfner MF (2001). Drosophila males transfer antibacterial proteins from their accessory gland and ejaculatory duct to their mates. J Insect Physiol.

[81] Ravi Ram K, Wolfner MF (2007). Seminal influences: *Drosophila* Acps and the molecular interplay between males and females during reproduction. Integr Comp Biol.

[82] Mack PD, Kapelnikov A, Heifetz Y, Bender M (2006). Mating-responsive genes in reproductive tissues of female *Drosophila melanogaster*. Proc Natl Acad Sci.

[83] Azambuja P, Feder D, Garcia ES (2004). Isolation of *Serratia marcescens* in the midgut of *Rhodnius prolixus*: Impact on the establishment of the parasite *Trypanosoma cruzi* in the vector. Exp Parasitol.

[84] Wallis RC, Lang CA (1956). Egg formation and oviposition in blood-fed *Aedes aegypti* L. Mosq News.

[85] Judson CL (1967). Feeding and oviposition behavior in the mosquito Aedes aegypti (L.). I. Preliminary studies of physiological control mechanisms. Biol Bull Marine.

[86] Zouache K, Voronin D, Tran-Van V, Mousson L, Failloux A-B, Mavingui P (2009). Persistent *Wolbachia* and cultivable bacteria infection in the reproductive and somatic tissues of the mosquito vector *Aedes albopictus*. PLoS ONE..

[87] Martinson VG, Magoc T, Koch H, Salzberg SL, Moran NA (2014). Genomic features of a bumble bee symbiont reflect its host environment. Appl Environ Microbiol.

[88] Lee FJ, Rusch DB, Stewart FJ, Mattila HR, Newton ILG (2015). Saccharide breakdown and fermentation by the honey bee gut microbiome. Environ Microbiol.

[89] White D (2000). Physiology and biochemistry of prokaryotes.

[90] Kuhn H-M, Meier-Dieter U, Mayer H (1988). ECA, the enterobacterial common antigen. FEMS Microbiol Rev.

[91] Ganley JG, Pandey A, Sylvester K, Lu K-Y, Toro-Moreno M, Rütschlin S (2020). A systematic analysis of mosquito-microbiome biosynthetic gene clusters reveals antimalarial siderophores that reduce mosquito reproduction capacity. Cell Chem Biol.

[92] Farrand SK, Taber HW (1973). Physiological effects of menaquinone deficiency in *Bacillus subtilis*. J Bacteriol.

[93] Farrand SK, Taber HW (1974). Changes in menaquinone concentration during growth and early sporulation in *Bacillus subtilis*. J Bacteriol.

[94] Knight R, Vrbanac A, Taylor BC, Aksenov A, Callewaert C, Debelius J (2018). Best practices for analysing microbiomes. Nat Rev Microbiol.

[95] Steven B, Hyde J, La Reau JC, Brackney DE (2021). The axenic and gnotobiotic mosquito: emerging models for microbiome host interactions. Front Microbiol.

