# Supplementary figures and images for: Characterization of the reproductive tract bacterial microbiota of virgin, mated, and blood-fed Aedes aegypti and Aedes albopictus females
Source: Parasit Vectors. 2021 Dec 1;14:592. doi: 10.1186/s13071-021-05093-7 (PMC8638121; doi:10.1186/s13071-021-05093-7)

A

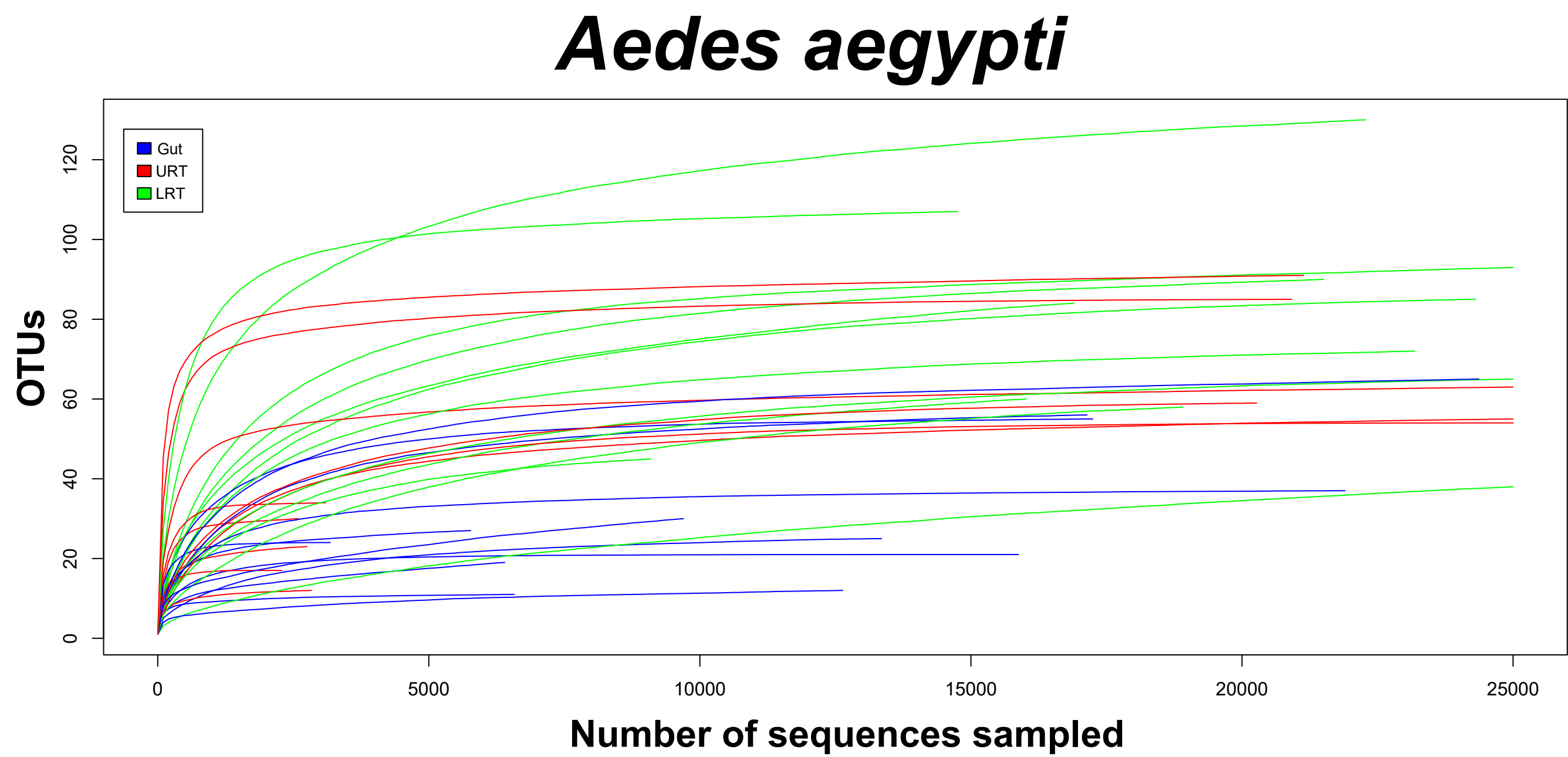

B

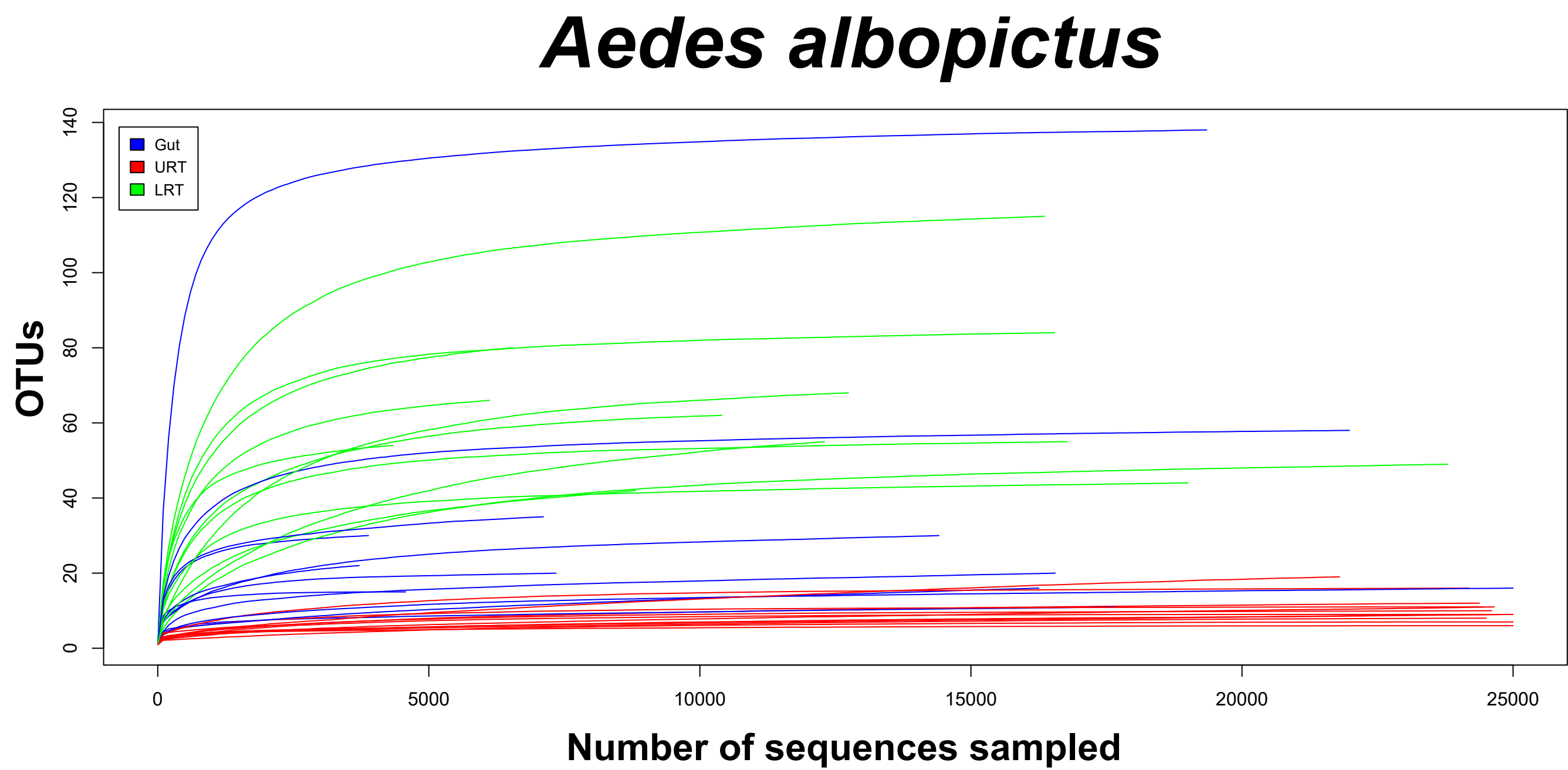

C

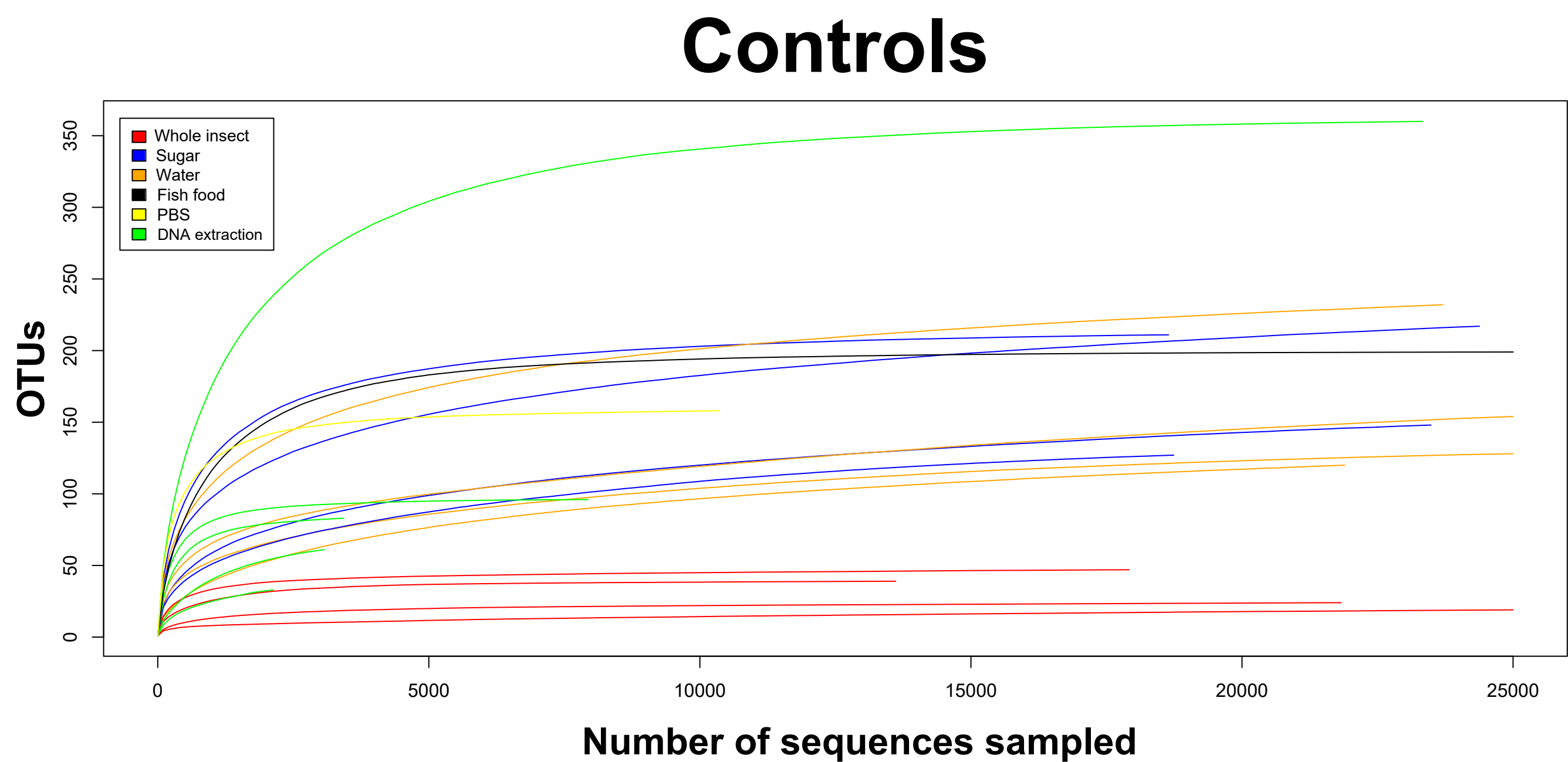

Supplement: Supplementary file 2 — Additional file 2: Figure S1. Rarefaction curves of tissue samples per host species and controls. [file 13071_2021_5093_MOESM2_ESM.pdf]

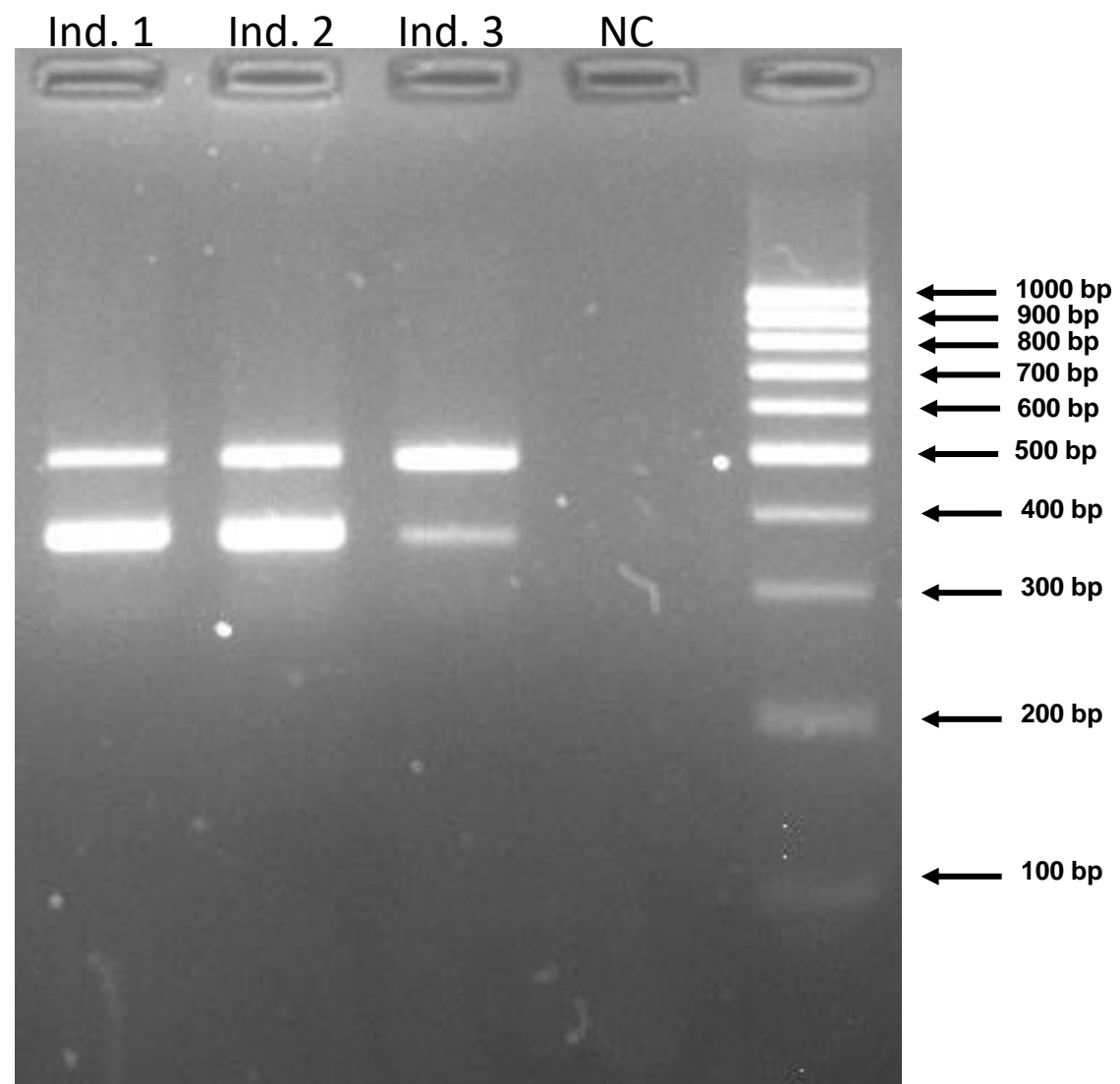

Supplement: Supplementary file 4 — Additional file 4: Figure S2. Agarose gel for three whole-body female Ae. albopictus from the Medellín strain laboratory colony. Lanes 1–3, individual samples; lane 4, negative control (NC); lane 5, molecular weight size marker. Band of 501 bp corresponds to wAlbB (Wolbachia supergroup B) and band of 379 bp to wAlbA (Wolbachia supergroup A). [file 13071_2021_5093_MOESM4_ESM.pdf]
