# Supplementary material for: Characterization of the reproductive tract bacterial microbiota of virgin, mated, and blood-fed Aedes aegypti and Aedes albopictus females
Source: Parasit Vectors. 2021 Dec 1;14:592. doi: 10.1186/s13071-021-05093-7 (PMC8638121; doi:10.1186/s13071-021-05093-7)
Supplement: Supplementary file 5 — Additional file 5: Figure S3. Relative OTUs abundance for control samples, virgin whole-body insects, larval rearing water, sucrose solution, food pellet, 1× PBS, and DNA extraction reagents. The 11 most abundant OTUs are shown, with those outside this group represented in the “other OTUs” category. [file 13071_2021_5093_MOESM5_ESM.pdf]

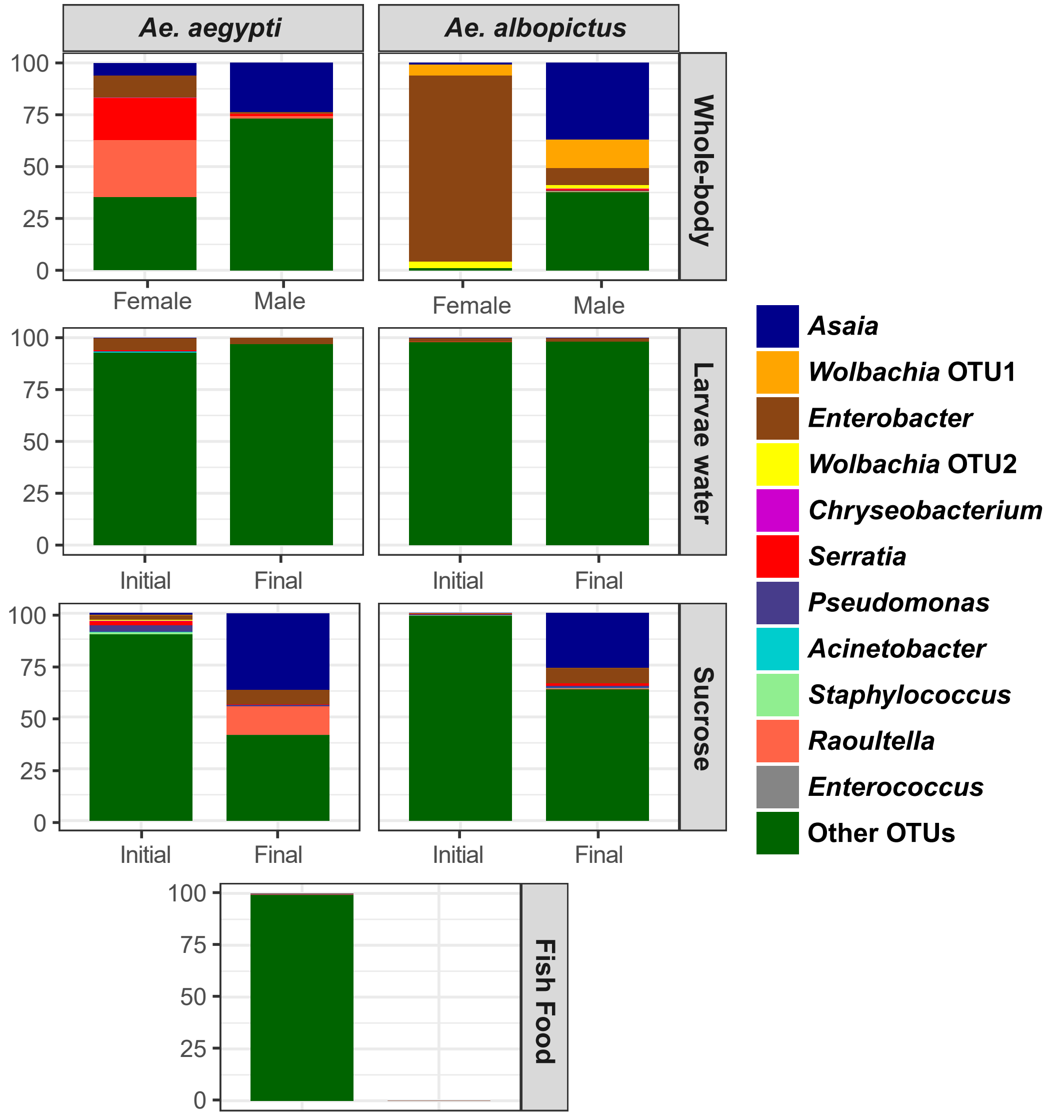

Ae. aegypti
Ae. albopictus
Whole-body
Larvae water
Sucrose
Fish Food
100
75
50
25
0
Female
Male
Initial
Final
Asaia
Wolbachia OTU1
Enterobacter
Wolbachia OTU2
Chryseobacterium
Serratia
Pseudomonas
Acinetobacter
Staphylococcus
Raoultella
Enterococcus
Other OTUs
